# Supplementary material for: Efficient Homology-Directed Repair with Circular Single-Stranded DNA Donors
Source: CRISPR J. 2022 Oct 13;5(5):685–701. doi: 10.1089/crispr.2022.0058 (PMC9595650; doi:10.1089/crispr.2022.0058)
Supplement: Supplemental data [file Suppl_FigS8.docx]

**Supplementary Fig. S8.**  Efficiency of fluorescent tag integration achieved with lssDNA donors generated using the TGIRT-mediated RT-PCR (T-lssDNA) or biotin-streptavidin affinity purification (B-lssDNA) approaches. Editing efficiencies for SpyCas9 RNPs and lssDNA donor delivery targeting the **(A)** *ACTB*, **(B)** *TOMM20*, **(C)** *SEC61B*, and **(D)** *GAPDH* loci in K562 (top panel) and HEK293T (bottom panel) cells are shown. Bars represent the mean from three independent biological replicates and error bars represent s.e.m.
