# Supplementary material for: Efficient Homology-Directed Repair with Circular Single-Stranded DNA Donors
Source: CRISPR J. 2022 Oct 13;5(5):685–701. doi: 10.1089/crispr.2022.0058 (PMC9595650; doi:10.1089/crispr.2022.0058)
Supplement: Supplemental data [file Suppl_FigS1.docx]

**Supplementary Fig. S1.** Preparation of different ssDNA templates. **(A)** Preparation of cssDNA: Donor DNA is cloned into phagemid vectors containing an f1 bacteriophage origin of replication and an antibiotic resistance marker. The plasmid is transformed into *E. coli* cells and superinfected with a helper phage. Depending on the orientation of the f1 origin, one particular strand is packaged into phage particles and extruded into the media from which phage particles are precipitated and cssDNA is purified. **(B)** Preparation of T-lssDNA: PCR product encoding donor DNA is generated using a 5’ primer containing a T7 promoter within the tail. The product is then used as a template for *in vitro* transcription to generate RNA. This RNA in turn is used as a template for reverse transcription using a reverse transcriptase such as TGIRT to generate linear ssDNA (T-lssDNA). **(C)** Preparation of B-lssDNA: A PCR primer is biotinylated at the 5’ end. The resulting biotinylated PCR product is then immobilized on streptavidin magnetic beads. The immobilized PCR product is then subjected to alkaline denaturation to separate the biotinylated strand from the non-biotinylated strand. The eluted non-biotinylated DNA strand is then recovered for use as lssDNA (B-lssDNA).
