# Supplementary material for: Efficient Homology-Directed Repair with Circular Single-Stranded DNA Donors
Source: CRISPR J. 2022 Oct 13;5(5):685–701. doi: 10.1089/crispr.2022.0058 (PMC9595650; doi:10.1089/crispr.2022.0058)
Supplement: Supplemental data [file Suppl_FigS2.docx]

**Supplementary Fig. S2.** Characterization of ssDNA. **(A)** 1% non-denaturing agarose gel image shows two representative T-lssDNA products targeting the *GAPDH* locus prior to gel purification of the correct product (indicated by “*”). **(B)** 1% non-denaturing agarose gel shows S1 nuclease digestion products of DNA templates. To determine whether the templates generated are entirely single-stranded, dsDNA products (Plasmid and PCR templates) and ssDNA templates (cssDNA, T-lssDNA and B-lssDNA) were digested with S1 nuclease. Undigested product (“Undig.") was loaded alongside S1 nuclease digested products (“Dig.”) and the gel was stained with GelRed stain from Biotium. **(C)** Sequencing analysis to detect the presence of duplex DNA templates. Sequencing chromatograms obtained using forward primer (left column, that can anneal to the ssDNA products) or the reverse primer (right column, that anneals to the complementary strand that should not be present in the cssDNA,T-lssDNA and B-lssDNA templates). The plasmid, T-lssDNA and B-lssDNA templates return the expected sequences that indicate the presence of a complementary strand within these DNA donor pools.
