# Supplementary material for: Efficient Homology-Directed Repair with Circular Single-Stranded DNA Donors
Source: CRISPR J. 2022 Oct 13;5(5):685–701. doi: 10.1089/crispr.2022.0058 (PMC9595650; doi:10.1089/crispr.2022.0058)
Supplement: Supplemental data [file Suppl_FigS3.docx]

**Supplementary Fig. S3.** Precise and imprecise editing efficacy of different Cas9 and Cas12a nucleases at the integrated TLR-MCV locus upon delivery of nucleases as plasmids. The graph depicts the percentage of mCherry-positive (shown in red, representative of the indel efficiency) and GFP-positive (shown in green, representative of the HDR efficiency) cells obtained after co-delivery of 250 ng plasmid-encoded nucleases, 250 ng of gRNA plasmid and 500 ng of plasmid donor DNA template into TLR-MCV1 HEK293T cells. Bars represent the mean from three independent biological replicates and error bars represent s.e.m.
