# Supplementary material for: Efficient Homology-Directed Repair with Circular Single-Stranded DNA Donors
Source: CRISPR J. 2022 Oct 13;5(5):685–701. doi: 10.1089/crispr.2022.0058 (PMC9595650; doi:10.1089/crispr.2022.0058)
Supplement: Supplemental data [file Suppl_FigS4.docx]

**Supplementary Fig. S4.** TIDE analysis to ascertain indel efficiencies at the integrated TLR-MCV1 locus in HEK293T cells. The graph shows indel percentages observed at the TLR-MCV1 locus using SpyCas9, LbaCas12a, AspCas12a and FnoCas12a effectors based on TIDE analysis of Sanger sequencing data from PCR amplicons spanning the locus following nuclease treatment (in the absence of donor DNA). The green bars show the percentage of insertions and the pink bars show the percentage of deletions. The data represent the mean from three biological replicates and error bars represent s.e.m.
