# Supplementary material for: Efficient Homology-Directed Repair with Circular Single-Stranded DNA Donors
Source: CRISPR J. 2022 Oct 13;5(5):685–701. doi: 10.1089/crispr.2022.0058 (PMC9595650; doi:10.1089/crispr.2022.0058)
Supplement: Supplemental data [file Suppl_FigS5.docx]

**Supplementary Fig. S5. (A)** Comparison of cssDNA- and T-lssDNA-mediated HDR efficiency upon treatment of TLR-MCV1 cells with distinct Cas effectors. The graphs depict the percentage of mCherry- and GFP-positive cells obtained after co-delivery of SpyCas9 or AspCas12a with cssDNA, T-lssDNA, B-ssDNA or plasmid DNA repair templates into TLR-MCV1 K562 cells (upper grey box) and TLR-MCV1 HEK293T cells (lower blue box). Bars represent the mean from three independent biological replicates and error bars represent the standard error of mean (s.e.m.). **(B)** The graphs depict the percentage of mCherry-(shown in red) and GFP-positive cells (shown in green) obtained after co-delivery of SpyCas9 with B-lssDNA and circularized B-lssDNA DNA repair templates into TLR-MCV1 K562 cells (upper grey box) and TLR-MCV1 HEK293T cells (lower blue box). Bars represent the mean from three independent biological replicates and error bars represent s.e.m. **(C)** Comparison of cssDNA- and T-lssDNA-mediated HDR efficiency upon treatment of TLR-MCV1 cells with distinct Cas effectors. The graphs depict the percentage of percentage of mCherry-(shown in red) and GFP-positive cells (shown in green) obtained after co-delivery of SpyCas9, AspCas12a, LbaCas12a or FnoCas12a with cssDNA and T-lssDNA DNA repair templates into TLR-MCV1 K562 cells (upper grey box) and TLR-MCV1 HEK293T cells (lower blue box). Bars represent the mean from three independent biological replicates and error bars represent s.e.m. **(D)** Effect of cssDNA and T-lssDNA donor orientation on HDR efficiency. The graphs depict the percentage of mCherry- and GFP-positive cells obtained after co-delivery of SpyCas9-1 or AspCas12a (targeting the same strand) with sense (S) and antisense (AS) strand cssDNA and T-lssDNA DNA repair templates into TLR-MCV1 K562 cells (upper grey box) and TLR-MCV1 HEK293T cells (lower blue box). Bars (GFP-positive cells in green & mCherry-positive cells in red) represent the mean from six independent biological replicates for HEK293T cells and three independent replicates for K562 Cells. Numbers above the bars indicate ratio of HDR to total editing events [*i.e.*, the number of GFP-positive cells divided by the total of mCherry-positive and GFP-positive cells (HDR ratio)]. Error bars represent s.e.m.
