# Supplementary material for: Efficient Homology-Directed Repair with Circular Single-Stranded DNA Donors
Source: CRISPR J. 2022 Oct 13;5(5):685–701. doi: 10.1089/crispr.2022.0058 (PMC9595650; doi:10.1089/crispr.2022.0058)
Supplement: Supplemental data [file Suppl_FigS6.docx]

**Supplementary Fig. S6.** Strand dependence of guide and HDR template on knock-in efficiency. **(A)** Schematic of guide (depicted by black and blue lines) and strand orientation relative to the TLR-MCV1 target site. The magenta triangles indicate the position of the SpyCas9 DSB. The green and red lines indicate GFP and mCherry encoding regions, respectively. The orange region depicts the small insertion containing target sites for Cas9 and Cas12a proteins. **(B)** The graphs depict the percentage of mCherry- and GFP-positive cells obtained after co-delivery of SpyCas9 complexed with guides (SpyCas9 RNP) targeting either strand of the TLR-MCV1 reporter along with DNA repair templates complementary to the antisense or sense strand in K562 cells (upper grey box) and HEK293T cells (lower blue box). Numbers above the bars indicate ratio of HDR to total editing events [*i.e.*, number of GFP-positive cells divided by the total of mCherry-positive and GFP-positive cells (HDR ratio)]. Bars represent the mean from three independent biological replicates for K562 cells and six independent replicates for HEK293T cells. Error bars represent s.e.m.
