# Supplementary material for: Efficient Homology-Directed Repair with Circular Single-Stranded DNA Donors
Source: CRISPR J. 2022 Oct 13;5(5):685–701. doi: 10.1089/crispr.2022.0058 (PMC9595650; doi:10.1089/crispr.2022.0058)
Supplement: Supplemental data [file Suppl_FigS7.docx]

**Supplementary Fig. S7**. SpyCas9 gene editing efficiency at the *ACTB*, *TOMM20*, *SEC61B* and *GAPDH* loci. Genome editing was achieved by electroporation of 20 pmoles SpyCas9 complexed with 25 pmoles of guide RNA into HEK293T cells in the absence of HDR donor. The editing percentages (indicated above the bars) were calculated by TIDE analysis of Sanger sequencing data from PCR amplicons spanning each locus following nuclease treatment. Pink bars indicate the percentage of deletions and green bars indicate the percentage of insertions.
