# Supplementary material for: Efficient Homology-Directed Repair with Circular Single-Stranded DNA Donors
Source: CRISPR J. 2022 Oct 13;5(5):685–701. doi: 10.1089/crispr.2022.0058 (PMC9595650; doi:10.1089/crispr.2022.0058)
Supplement: Supplemental data [file Suppl_FigS9.zip › Suppl_FigS9.docx]

**Supplementary Fig. S9.** Effect of orientation of cssDNA on HDR efficiency at endogenous loci. Editing efficiencies for SpyCas9 RNPs and cssDNA donors targeting the **(A)** *ACTB*, **(B)** *TOMM20*, **(C)** *SEC61B* and **(D)** *GAPDH* loci in K562 cells (top panel) and HEK293T cells (bottom panel) are shown. Green bars indicate the percentage of cells expressing GFP and red bars indicate the percentage of cells expressing iTAG-RFP/dTomato. Solid bars correspond to donor DNA in orientation 1 (ssDNA complementary to the antisense strand of the target gene) and hashed bars correspond to orientation 2 (ssDNA complementary to the sense strand of the target gene). Bars represent the mean from three independent biological replicates and error bars represent s.e.m.
