# Supplementary material for: Efficient Homology-Directed Repair with Circular Single-Stranded DNA Donors
Source: CRISPR J. 2022 Oct 13;5(5):685–701. doi: 10.1089/crispr.2022.0058 (PMC9595650; doi:10.1089/crispr.2022.0058)
Supplement: Supplemental data [file Suppl_FigS10.docx]

**Supplementary Fig. S10**. Confocal images showing insertion of GFP or iTAG-RFP at the *ACTB* locus (top panel); GFP or dTomato at the *SEC61B* locus (middle panel), or GFP or dTomato at the *TOMM20* locus (bottom panel) in HEK293T cells from the experiments shown in Figure 3.
