# Supplementary material for: Efficient Homology-Directed Repair with Circular Single-Stranded DNA Donors
Source: CRISPR J. 2022 Oct 13;5(5):685–701. doi: 10.1089/crispr.2022.0058 (PMC9595650; doi:10.1089/crispr.2022.0058)
Supplement: Supplemental data [file Suppl_FigS11.docx]

**Supplementary Fig. S11.** Biallelic integration of GFP and iTagRFP in K562 cells using cssDNA templates. K562 cells were electroporated with 1 pmol each of GFP- and iTagRFP-encoding cssDNA templates along with 20 pmols of SpyCas9 complexed with 25 pmols of guide RNA targeting *ACTB*. The green bar represent the percentage of GFP-positive cells, the red bar represents iTagRFP-expressing cells and the yellow bar represents cells expressing both GFP and iTagRFP. Bars represent the mean from three independent biological replicates and error bars represent s.e.m.
