# Supplementary material for: Efficient Homology-Directed Repair with Circular Single-Stranded DNA Donors
Source: CRISPR J. 2022 Oct 13;5(5):685–701. doi: 10.1089/crispr.2022.0058 (PMC9595650; doi:10.1089/crispr.2022.0058)
Supplement: Supplemental data [file Suppl_FigS12.docx]

**Supplementary Fig. S12.** Lengths of cssDNA that can be generated efficiently. 1% non-denaturing agarose gel image showing 1kb ladder (lane 1), or cssDNA generated from plasmids that are 5.4kb (lane 2), 6.2kb (lane 3), 8.2kb (lane 4) and 13.6 kb (lane 5) in length.
